# Dietary supplementation with daidzein and Chinese herbs, independently and combined, improves laying performance, egg quality and plasma hormone levels of post-peak laying hens

**DOI:** 10.1016/j.psj.2021.101115

**Published:** 2021-03-12

**Authors:** Ling Zhang, Guang Zhong, Wenjie Gu, Na Yin, Long Chen, Shourong Shi

**Affiliations:** ⁎ Jiangsu Agri-animal Husbandry Vocational College, Taizhou, Jiangsu Province, PR China, 225300; † Poultry Institute, Chinese Academy of Agricultural Sciences, Yangzhou, Jiangsu Province, PR China, 225125; ‡ Jiangsu Co-Innovation Center for the Prevention and Control of Important Animal Infectious Disease and Zoonosis, Yangzhou, Jiangsu Province, PR China, 225009

**Keywords:** daidzein, Chinese herb, post-peak laying hen, laying performance, egg quality

## Abstract

This experiment examined the separate and combined effects of daidzein (**Da**) and Chinese herbs (**CH**) on laying performance and egg quality of post-peak laying hens. Additionally, we explored potential mechanisms of action for these 2 additives by examining plasma hormone levels. After 4 wk of acclimation to caging, 60-week-old Hyline Brown hens (360) were selected and randomly divided into 4 groups with 6 replicates and 15 chickens per replicate. The following 4 dietary groups were utilized: 1) control group (basal diet); 2) Da group (basal diet + 0.03 kg/t DA); 3) CH group (basal diet +0.6 kg/t CH); 4) Da + CH group (basal diet + 0.03 kg/t Da + 0.6 kg/t CH). Data were analyzed in a completely randomized design with a 2×2 factorial arrangement of treatments. Egg production and FCR treatment averages were analyzed in the following 3 phases: wk 1-4, 5-8, and 1-8 of treatment administration. Results revealed that Da increased egg production but decreased FCR (*P <* 0.05) for wk 1-8 and especially during wk 5-8 (*P <* 0.05). CH decreased FCR in wk 1-4 and 5-8 (*P <* 0.05), but increased egg production only during wk 5-8 (*P <* 0.05). Da increased Haugh units (*P <* 0.05) on wk 4 and 8; CH increased Haugh units (*P <* 0.05) but decreased yolk ratio (*P <* 0.05) on wk 4 and 8. Da increased the plasma levels of T3, PROG, FSH, LH and E2 (*P <* 0.05); CH increased the plasma level of T3 (*P <* 0.05). Additionally, Da x CH interactions existed for albumen height, Haugh units, albumen ratio and the level of T3 on wk 8 (*P <* 0.05), indicating that the combination of Da and CH was more effective than administration of either of these dietary components independently. In conclusion, Da and CH, both independently and in combination, increase laying performance, egg quality and plasma hormones levels in post-peak laying hens. Therefore, these treatments may be able to provide prolonged economic benefits to aged laying hens.

## INTRODUCTION

Daidzein (**Da**), a flavonoid found in soybeans, red clover, and kudzu root (Messina and Gleason, 2016), structurally resembles 17-β-oestradiol (Xiao et al., 2018). In addition to the unique antioxidant function of flavonoids, Da is estrogen-like and exhibits estrogenic behavior by binding to estrogen receptors in animals, humans and cultured cells (Hong et al., 2004; Liu et al., 2006; Xiao et al., 2018). Lu et al (2017) found that egg production was improved after giving 200 mg/kg Da to laying hens, and Tang et al. (2006) found that Da exhibited an antioxidant function during primordial germ (egg) cell proliferation. Therefore, Da appears to be a potential treatment to improve animal reproductive performance.

Additionally, in recent years, Chinese herbs (**CH**) have been tested as dietary supplements to improve animal performance (Wu, 2018). For over 5,000 yr, CH have been used in China. In the book *Classical Chinese Pharmacopoeia* (Li et al., 2015), numerous CH formulations were described to treat diseases and sustain health. For example, *Radix Astragali*, a perennial herb, has numerous beneficial effects, such as enhancing immune and antioxidant function (Lu et al., 2016). *Salvia miltiorrhiza Bunge* has been used to treat various diseases, such as cancer and bone loss (Su et al., 2015). *Cnidium monnieri* plays effective roles in immune and antioxidant functions as well as in reducing the response to stress (Li et al., 2016). The effects of Chinese herbs on promoting reproduction have also been confirmed (Jiang et al., 2019). Perhaps mixing some of these herbs in specific proportions could exert greater effects than even modern drugs. Additionally, it is very important to remember that even some of the modern-day drugs have been derived from plants, i.e., salicylic acid (aspirin) from willow bark (Shara and Stohs, 2015).

As is well known, estrogen is proposed to be a pivotal factor involved in development and maintenance of the female reproductive system and ovum formation, and vitellogenesis is one of the key factors necessary for egg production (Liu and Zhang, 2008). However, the level of estrogen decreases gradually with age resulting in a decline in laying performance in post-peak laying hens. Thus, raising the level of estrogen may be a method to increase laying performance in post-peak laying hens. It has been reported that Da is becoming increasingly popular as a dietary supplement, particularly for post-peak-estrus animals, possibly, in part, because of it estrogenic properties (Shi et al., 2013). Some scholars have also used CH as feed additives and improved animal production (Wang et al., 2019). In 2019, Xiao et al. revealed that a dietary mixture of CH and Da improved laying performance and plasma antioxidant activity as well as LH levels and mineral content as compared to a control diet. However, that experiment did not examine the independent effects of Da and CH to determine their separate vs combined effects. Therefore, in the present study, the separate as well as combined effects of Da and CH on laying performance and egg quality in post-peak laying hens were examined in a 2×2 factorial arrangement of dietary treatments. Additionally, potential mechanisms of action for these 2 additives were explored by examining plasma hormone levels.

## MATERIALS AND METHODS

### Experimental Design, Birds, and Dietary Treatments

After 4 wk of acclimation to caging and a basal diet, 60-wk-old Hyline Brown hens (post-peak egg production) with similar laying rates were selected and randomly divided into 4 groups with 6 replicates and 15 chickens per replicate (360 hens total). A 2×2 factorial arrangement of dietary treatments was utilized to create the following 4 treatment groups: 1) control group (basal diet); 2) Da group (basal diet + 0.03 kg/t Da); 3) CH group (basal diet + 0.6 kg/t CH); 4) Da + CH group (basal diet + 0.03 kg/t Da + 0.6 kg/t CH). The Da (10% purity, adding 0.3kg/t to the diet to ensure the active ingredient is 0.03kg/t) was purchased from Sichuan Guanghan Feed Co. Ltd., Guanghan, Sichuan Province, PR China. CH was a mixture of *Radix Astragali, Salvia miltiorrhiza Bunge,* and *Cnidium monnieri* (purchased from Jingui Traditional Chinese Medicine Co. Ltd, Wuhan, Hubei Province, PR China). The ratio of *Radix Astragali: Salvia miltiorrhiza Bunge: Cnidium monnieri* was 1:1:1. This experiment lasted for 8 wk.

### Management

This experiment was approved by the Animal Care and Use Committee of Jiangsu Agri-animal Husbandry Vocational College (Taizhou, Jiangsu Province, PR China). The experiment uses three layers of cages in close sided house, and one bird in each cage. The size of the cage is 25 cm * 35 cm, so that the density is guaranteed to be about 0.09 m^2^/bird. Birds had free access to feed and water, the photoperiod was set at 16 L: 8 D throughout the study, and ventilation measures were the same as the HY-Line layer breeding standards. Basal diets were formulated to meet or exceed NRC (1994) recommendations. The composition and nutrient levels of the basal diet are shown in Table 1.

**Table 1:** Composition and nutrient levels of the basal diet (air dry basis).

Ingredients	Content (%)
Corn	62.70
Soybean (43%)	26.30
CaHPO_4_	1.00
*DL*-Met	0.10
CaCO_3_	8.50
Choline chloride (50%)	0.1
NaCl	0.3
Vitamin and trace mineral Premix^1^	1.00
Total	100.00
Nutrient levels^2^	
ME (MJ/kg)	11.09
CP	16.61
Ca	3.5
NPP	0.35
D Lys	0.85
Met	0.35

1 Premix provided per kilogram of diet: vitamin A (retinyl palmitate): 7715 IU; vitamin D_3_ (cholecalciferol): 2755 IU; vitamin E (dl-atocopheryl acetate): 8.8 IU; vitamin K_3_ (menadione sodium bisulfate complex): 2.2 mg; vitamin B_12_ (cobalamin), 0.01 mg; menadione (menadione sodium bisulfate complex): 0.18 mg; riboflavin: 4.41 mg; pantothenic acid (d-calcium pantothenate): 5.51 mg; niacin: 19.8 mg; folic acid: 0.28 mg; pyridoxine (pyridoxine hydrochloride): 0.55 mg; Mn (manganese sulfate): 50 mg; Fe (ferrous sulfate): 25 mg; Cu (copper sulfate): 2.5 mg; Zn (zinc sulfate): 50 mg; I (calcium iodate): 1.0 mg; and Se (sodium selenite): 0.15 mg.

2 Metabolizable energy is calculated, whereas all other values are analyzed.

### Sample Collection and Analytical Determination

***Laying Performance.*** During this experimental stage, the number of eggs and laying hens as well as egg weight and feed intake were recorded daily. Egg production was expressed as average hen-day production, which was the total eggs laid divided by the total number of laying hens alive during the period and the total number of days in the period. Average egg weight was calculated as total egg weight divided by the number of eggs. Average daily feed intake (ADFI) was calculated once a week, and feed conversion ratio (FCR) was represented through the ratio of total feed consumption with total egg weight at every stage.

***Egg Quality.*** At the end of wk 4 and 8, 72 eggs (3 eggs/each replicate) were selected randomly, and egg quality was measured. The egg quality characteristics included egg weight, shape index, albumen height, Haugh units, yolk color, and yolk weight as well as eggshell weight, thickness, and strength. The ratios of shell, yolk and albumen were then calculated relative to whole egg weight.

Longitudinal and transverse egg diameters were measured with a digital caliper (Guanglu Measuring Instrument Co., Ltd, Guilin, Guangxi Province, PR China), and the shape index was expressed by the ratio of longitudinal diameter to transverse diameter. Eggshell thickness was measured and recorded once at the air cell end, equator and small end of each egg with an Egg Shell Thickness Gauge (ESTG-1 type, ORKA Food Technology Ltd. Ramat Hasharon, Israel) and then averaged together for each egg. Eggshell strength was measured with an Egg Force Reader (ORKA Food Technology Ltd., Ramat Hasharon, Israel). Haugh units, albumen height and yolk color were measured with a multi-function egg quality tester (EA-01 type, ORKA Food Technology Ltd., Ramat Hasharon, Israel).

After the aforementioned measurements were taken, the yolk was separated and weighed. The eggshell was weighed after cleaning and drying, and the mass of albumen was calculated as the difference in egg weight minus yolk and shell weight.

***Plasma Hormones.*** At the end of this study, 24 blood samples (one sample per replicate) were collected from brachial vein, and allowed to clot for 30 to 60 min before plasma was separated. The levels of triiodothyronine (T3), thyroxine (T4), progesterone (PROG), estrogen (E2), follicle-stimulating hormone (FSH) and luteinizing hormone (LH) in plasma were measured by radioimmunoassay using commercial kits (Beifang Biotech Research Institute, Beijing, PR China).

### Statistical Analysis

A two-way ANOVA was used to examine responses to Da and CH supplementation and their interactions in a completely randomized design with a 2×2 factorial arrangement of treatments. All data were analyzed by the general linear model (GLM) procedure of SPSS (SPSS 20.0, IBM Inc., New York, US) software, and differences were assumed to be statistically significant when *P <* 0.05. From treatment averages obtained across weeks of the study, egg production as well as feed intake and conversion data were analyzed in the following 3 phases: wk 1-4, 5-8, and 1-8. The 6 replicates of laying hens per treatment were the experimental units for all data.

## RESULTS

### Laying Performance

Laying performance is shown in Tables 2 to 4. CH increased egg production in wk 5-8 (*P =* 0.047) but decreased FCR of laying hens in wk 1-4 (*P =* 0.035) and 5-8 (*P =* 0.024). In wk 5-8, Da increased egg production (*P =* 0.031) but decreased FCR (*P =* 0.036). Overall from wk 1-8, Da increased egg production (*P =* 0.048) and decreased FCR (*P =* 0.044). However, there were no differences detected for egg weight or ADFI (*P >* 0.05), and no Da x CH interactions were detected for any laying performance characteristic (*P >* 0.05).

**Table 2:** Impact of Daidzein (Da) and Chinese herbs (CH) on laying performance of post-peak laying hens during wk 1-4 of treatment.

Factors		Performance characteristic			
Da	CH	Egg weight (g)	Egg production (%)	ADFI (g)	FCR
Interaction means					
-	-	66 ± 1.8	82 ± 3.0	123 ± 6.7	2.3 ± 0.15
	+	66 ± 1.6	87 ± 4.4	120 ± 9.0	2.1 ± 0.14
+	-	67 ± 1.2	86 ± 3.0	123 ± 3.8	2.2 ± 0.13
	+	67 ± 1.3	89 ± 3.8	127 ± 5.7	2.1 ± 0.12
Main effect means					
-		66.1	84.3	121.4	2.20
+		67.1	87.5	124.5	2.16
	-	66.6	83.7	123.3	2.21^a^
	+	66.7	88.0	122.6	2.14^b^
ANOVA					
*P*_Da_		0.143	0.082	0.272	0.054
*P*_CH_		0.879	0.075	0.103	0.035
*P*_Da*CH_		0.810	0.112	0.116	0.104

^a-b^Main effect means with different letters are significantly different (*P <* 0.05); N = 6, data are expressed as mean ± SE.

**Table 3:** Impact of daidzein (Da) and Chinese herbs (CH) on laying performance of post-peak laying hens during wk 5-8 of treatment.

Factors		Performance characteristic			
Da	CH	Egg weight (g)	Egg production (%)	ADFI (g)	FCR
Interaction means					
-	-	66 ± 1.4	81 ± 2.5	120 ± 3.8	2.4 ± 0.08
	+	65 ± 1.4	84 ± 6.5	123 ± 7.0	2.2 ± 0.11
+	-	67 ± 1.7	87 ± 2.6	120 ± 6.4	2.1 ± 0.14
	+	66 ± 1.8	89 ± 3.8	120 ± 5.7	2.1 ± 0.15
Main effect means					
-		65.8	82.4^b^	123.0	2.28^a^
+		66.4	87.6^a^	120.0	2.13^b^
	-	66.5	83.7^b^	119.8	2.26^a^
	+	65.7	86.5^a^	121.5	2.15^b^
ANOVA					
*P*_Da_		0.323	0.031	0.475	0.036
*P*_CH_		0.220	0.047	0.394	0.024
*P*_Da*CH_		0.884	0.120	0.149	0.783

^a-b^Main effect means with different letters are significantly different (*P <* 0.05); N = 6, data are expressed as mean ± SE.

**Table 4:** Impact of Daidzein (Da) and Chinese herbs (CH) on laying performance of post-peak laying hens during wk 1-8 of treatment.

Factors		Performance characteristics			
Da	CH	Egg weight (g)	Egg production (%)	ADFI (g)	FCR
Interaction means					
-	-	66 ± 1.5	81 ± 2.8	122 ± 4.4	2.3 ± 0.12
	+	66 ± 1.5	84 ± 5.4	121 ± 6.5	2.2 ± 0.15
+	-	67 ± 1.7	86 ± 2.7	121 ± 4.4	2.2 ± 0.10
	+	68 ± 1.6	88 ± 3.7	123 ± 4.2	2.1 ± 0.13
Main effect means					
-		66.4	83.3^b^	121.5	2.30^a^
+		66.8	87.6^a^	122.9	2.15^b^
	-	66.5	83.9	121.6	2.24
	+	67.2	86.1	122.8	2.18
ANOVA					
*P*_Da_		0.585	0.048	0.489	0.044
*P*_CH_		0.820	0.137	0.664	0.385
*P*_Da*CH_		0.798	0.520	0.543	0.741

^a-b^ Main effect means with different letters are significantly different (*P <* 0.05); N = 6, data are expressed as mean ± SE.

### Egg Quality

Egg quality characteristics are shown in Tables 5 and 6. On wk 4, Da (*P =* 0.033) and CH (*P =* 0.042) increased Haugh units. During this same week CH also increased albumen ratio (*P =* 0.011) but decreased yolk ratio (*P =* 0.017). On wk 8, Da (*P =* 0.049) and CH (*P =* 0.047) again increased Haugh units; however, an interaction revealed that the combination of Da and CH yielded the greatest increase in Haugh units (*P* = 0.006). On wk 8, CH also decreased yolk ratio (*P =* 0.017). Additionally, interactions revealed that the combination of Da and CH was most effective at increasing albumen height (*P =* 0.025) and albumen ratio (*P =* 0.018).

**Table 5:** Daidzein (Da) and Chinese herbs (CH) increase egg quality of post-peak laying hens on wk 4 of treatment.

Factor		Egg quality characteristic								
Da	CH	Shape index	Shell thickness (mm)	Shell strength (N)	Albumen height (mm)	Yolk color	Haugh unit	Albumen ratio (%)	Yolk ratio (%)	Shell ratio (%)
Interaction means										
-	-	1.30 ± 0.022	0.38 ± 0.035	29 ± 3.0	7.9 ± 0.95	7.1 ± 0.40	78 ± 6.1	61.9 ± 2.16	27.4 ± 1.46	10.7 ± 0.83
	+	1.31 ± 0.021	0.36 ± 0.034	29 ± 3.3	7.4 ± 0.52	7.2 ± 0.14	81 ± 3.7	63.7 ± 0.59	25.9 ± 0.77	10.4 ± 0.72
+	-	1.31 ± 0.024	0.37 ± 0.042	28 ± 2.4	7.7 ± 0.77	7.3 ± 0.42	81 ± 3.5	62.0 ± 1.04	26.9 ± 1.28	10.9 ± 0.80
	+	1.30 ± 0.017	0.37 ± 0.029	29 ± 3.1	7.8 ± 0.48	7.2 ± 0.39	83 ± 3.0	63.6 ± 1.23	25.9 ± 1.00	10.4 ± 0.49
Main effect means										
-		1.312	0.371	29.2	7.66	7.15	79.6^b^	62.78	26.66	10.54
+		1.308	0.372	28.5	7.72	7.24	82.7^a^	62.78	26.40	10.66
	-	1.311	0.378	28.8	7.78	7.20	79.3^b^	61.93^b^	27.17^a^	10.79
	+	1.304	0.374	28.9	7.60	7.18	82.0^a^	63.63^a^	25.89^b^	10.41
ANOVA										
*P*_Da_		0.654	0.831	0.793	0.853	0.548	0.033	0.996	0.604	0.725
*P*_CH_		0.826	0.436	0.983	0.554	0.866	0.042	0.011	0.017	0.834
*P*_Da*CH_		0.305	0.654	0.909	0.354	0.501	0.081	0.834	0.617	0.878

^a-b^Main effect means with different letters are significantly different (*P <* 0.05); N = 6, data are expressed as mean ± SE.

**Table 6:** Daidzein (Da) and Chinese herbs (CH) increases egg quality of post-peak laying hens on wk 8 of treatment.

Factor		Egg quality characteristic								
Da	CH	Shape index	Shell thickness (mm)	Shell strength (N)	Albumen height (mm)	Yolk color	Haugh unit	Albumen ratio (%)	Yolk ratio (%)	Shell ratio (%)
Interaction means										
-	-	1.30 ± 0.032	0.39 ± 0.016	28 ± 1.2	6.2 ± 0.58^b^	6.9 ± 0.24	72 ± 2.3^c^	62.8 ± 0.90^ab^	27.0 ± 1.12	10.2 ± 0.64
	+	1.32 ± 0.031	0.39 ± 0.013	34 ± 1.3	6.6 ± 0.29^ab^	7.0 ± 0.22	79 ± 1.7^ab^	62.6 ± 1.31^ab^	26.6 ± 1.25	10.7 ± 0.62
+	-	1.32 ± 0.028	0.40 ± 0.026	31 ± 1.3	6.7 ± 0.14^ab^	6.9 ± 0.33	77 ± 2.2^b^	61.7 ± 0.75^b^	27.3 ± 0.87	10.8 ± 0.56
	+	1.30 ± 0.033	0.39 ± 0.025	32 ± 1.1	6.9 ± 0.09^a^	6.9 ± 0.29	80 ± 1.4^a^	63.7 ± 1.23^a^	25.4 ± 0.96	10.8 ± 0.48
Main effect means										
-		1.314	0.395	31.1	6.38	6.92	76.3^b^	62.75	26.83	10.50
+		1.315	0.392	31.8	6.81	6.90	79.2^a^	62.74	26.33	10.68
	-	1.315	0.396	29.9	6.52	6.93	76.4^b^	62.38	27.10^a^	10.52
	+	1.316	0.394	33.1	6.79	6.96	79.5^a^	63.16	26.10^b^	10.78
ANOVA										
*P*_Da_		0.657	0.642	0.672	0.716	0.892	0.049	0.922	0.314	0.435
*P*_CH_		0.771	0.780	0.239	0.215	0.711	0.047	0.056	0.017	0.094
*P*_Da*CH_		0.136	0.705	0.338	0.025	0.711	0.006	0.018	0.098	0.538

^a-b^Interaction and main effect means with different letters are significantly different (*P <* 0.05); N = 6, data are expressed as mean ± SE.

### Plasma Hormones

Plasma hormone levels are shown in Table 7. Da increased the level of T3 (*P =* 0.039), PROG (*P =* 0.024), FSH (*P =* 0.039), LH (*P =* 0.032) and E2 (*P =* 0.047); CH increased the level of T3 (*P =* 0.048). However, an interaction indicated that the combination of Da and CH yielded the greatest increase in the level of T3 (*P =* 0.045).

**Table 7:** Daidzein (Da) and Chinese herbs (CH) increases plasma hormone levels of post-peak laying hens on wk 8 of treatment.

Factor		Plasma hormone levels					
Da	CH	T3 (ng/mL)	T4 (ng/mL)	PROG (ng/mL)	FSH (mIU/mL)	LH (mIU/mL)	E2 (pg/mL)
Interaction means							
-	-	2.6 ± 0.36^c^	7 ± 1.1	0.8 ± 0.15	1.1 ± 0.03	0.11 ± 0.006	327 ± 10.5
	+	2.9 ± 0.42^b^	7 ± 1.3	0.9 ± 0.17	1.2 ± 0.04	0.13 ± 0.010	373 ± 13.6
+	-	3.4 ± 0.24^b^	6 ± 0.9	1.0 ± 0.38	1.3 ± 0.06	0.15 ± 0.009	404 ± 16.4
	+	4.0 ± 1.02^a^	7 ± 0.9	1.0 ± 0.37	1.3 ± 0.05	0.13 ± 0.101	384 ± 18.3
Main effect means							
-		2.75^b^	7.1	0.85^b^	1.17^b^	0.121^b^	351.3^b^
+		3.65^a^	6.6	1.02^a^	1.31^a^	0.143^a^	394.3^a^
	-	3.02^b^	6.2	0.91	1.23	0.136	365.5
	+	3.44^a^	7.5	0.98	1.25	0.132	379.1
ANOVA							
*P*_Da_		0.039	0.494	0.024	0.032	0.048	0.047
*P*_CH_		0.048	0.108	0.859	0.286	0.720	0.073
*P*_Da*CH_		0.045	0.519	0.505	0.475	0.721	0.749

^a-b^Interaction or main effect means with different letters are significantly different (*P <* 0.05); N = 6, data are expressed as mean ± SE.

Abbreviations: T3, triiodothyronine; T4, thyroid hormone; PROG, progesterone; FSH, follicle-stimulating hormone; LH, luteinizing hormone; E2, estradiol.

## DISCUSSION

### Laying Performance

From a production point of view, it is of great economic significance to study the effect of Da on laying performance, especially because egg production declines in post-peak laying hens. At present, controversy exists about the effect of Da on laying performance. For example, dietary supplementation of 200 g/t Da has increased egg production and egg weight (Xiao et al., 2019). Shi et al. (2013) also reported that various doses of Da could increase laying performance of post-peak laying hens. However, Zhao et al. (2005) indicated that the effects of Da were dependent upon species and physiological conditions as well as doses or durations of administration which could lead to positive, negative or no effects of Da on laying performance. In the present study, we found a positive effect of dietary Da on egg performance of post-peak laying hens, which is similar to the results of previous studies (Cai, et al. 2013; Gu et al., 2013; Shi, et al., 2013). We also found that adding Da into the diet for more than 1 mo, yielded the greatest improvement in laying performance of post-peak laying hens. Additionally, our results revealed that feeding Da alone also decreases feed consumption in post-peak laying hens, which has not been found in previous studies. One possible reason for these improvements might be related to the antioxidant effects of flavonoids, which might play an effective role in NF-κB-activation (Zhang et al., 2019).

CH have also been used as feed additives in recent years with some positive effects. For example, Zhou et al. (2009) found that CH increase laying rate. Li et al. (2005) reported that dietary supplementation with *Ligustrum lucidum or Schisandra chinensis* increases egg production of laying hens. Xiao et al. (2019) also found positive effects of CH on egg production. In the present study, CH also increased egg production in post-peak laying hens. Additionally, feed consumption was reduced in the current study by CH; however, due to the complex composition and action pathways of CH, it is difficult to explain the mechanisms responsible for these improvements.

### Egg Quality

The egg is the most important product of commercial layer production; therefore, egg quality is crucial in evaluating production efficiency, both in commerce and in research. As hens age past the peak period of lay, egg quality decreases rapidly (Rodriguez-Navarro et al., 2002; Bain et al., 2016). However, dietary Da has increased egg quality during the late laying period of quail (Sahin et al., 2007). Cai et al (2013) also found that eggshell thickness and eggshell strength were increased linearly with increasing dietary Da supplementation. However possibly due to the complex composition of CH, literature is lacking on the impact of CH on egg quality. In the present study, we found that Da increases Haugh units, and CH increases both Haugh units and albumen ratio, indicating that Da and CH can both independently increase egg quality. Interestingly, the dietary combination of Da and CH led to the greatest increases in albumen height, Haugh units and albumen ratio. However, the present study did not reveal any differences in shell thickness or shell strength due to treatment, which is unlike the results of Xiao et al (2019) when Da and CH were fed in combination. One possible reason for the lack of a response to dietary treatment for these 2 shell characteristics in the present study might be related to the much higher dietary dosage of Da utilized in the present study (0.03% Da) vs. the 2019 study (0.0059% Da).

### Plasma Hormones

As is well known, estrogen is a major regulator of female reproductive development in poultry. In fact, a dose dependent relationship exists between plasma E2 and oviduct growth and function (Klandorf et al., 1992; Qin et al., 1993). Therefore in laying hens, the intrinsic estrogenic state is reflected in the level of E2 in plasma. The level of plasma E2 is, to some extent, indicative of ovarian follicular development; and ovarian follicular development is predictive of future egg production (Tanabe et al., 1979; Leszczynski et al., 1985). It has also been reported that thyroid hormones play a role in the regulation of uterine responses to estradiol (Bottazzi et al., 1996), and the level of T3 in plasma is associated with oviduct development (Vermaut et al., 1998). The Chinese herb, *Cnidium monnieri*, has been shown to improve E2 plasma levels in female rats (Li et al., 2015). Setchell and Cassidy (1999) found that when endogenous E2 concentrations were low in vivo, Da might serve as an agonist to occupy vacant estrogen-binding sites resulting in an increase of the systemic estrogenic effect. Xiao et al (2019) reported that the dietary combination of Da and CH increased LH levels and thereby increased egg production. In the present study, Da alone increased the levels of T3, PROG, LH and E2; and CH alone increased T3 levels. However also in the present study, the dietary combination of Da and CH led to the greatest increase in T3 levels, indicating a synergistic effect of these 2 dietary elements on T3. These data confirm that Da and CH can increase laying performance and egg quality by increasing plasma hormone levels.

In conclusion, Da and CH, both independently and in combination can increase laying performance, egg quality and plasma hormones levels in post-peak laying hens. These improvements can lead to prolonged economic benefits of aged laying hens.
